# Temporal trends of care practices, morbidity, and mortality of extremely preterm infants over 10-years in South Wales, UK

**DOI:** 10.1038/s41598-020-75749-4

**Published:** 2020-10-30

**Authors:** Lieve Boel, Sujoy Banerjee, Megan Clark, Annabel Greenwood, Alok Sharma, Nitin Goel, Gautam Bagga, Chuen Poon, David Odd, Mallinath Chakraborty

**Affiliations:** 1 Regional Neonatal Intensive Care Unit, University Hospital of Wales, Cardiff, CF14 4XW UK; 2 Neonatal Intensive Care Unit, Singleton Hospital, Swansea, UK; 3 School of Medicine, Cardiff University, Cardiff, UK; 4 Neonatal Intensive Care Unit, Royal Gwent Hospital, Newport, UK; 5 Division of Population Medicine, School of Medicine, Cardiff University, Cardiff, UK; 6 Centre for Medical Education, School of Medicine, Cardiff University, Cardiff, UK

**Keywords:** Health care, Paediatrics, Neonatology

## Abstract

Contemporary outcome data of preterm infants are essential to commission, evaluate and improve healthcare resources and outcomes while also assisting professionals and families in counselling and decision making. We analysed trends in clinical practice, morbidity, and mortality of extremely preterm infants over 10 years in South Wales, UK. This population-based study included live born infants < 28 weeks of gestation in tertiary neonatal units between 01/01/2007 and 31/12/2016. Patient characteristics, clinical practices, mortality, and morbidity were studied until death or discharge home. Temporal trends were examined by adjusted multivariable logistic regression models and expressed as adjusted odds ratios (aOR) with 95% confidence intervals (95% CI). A sensitivity analysis was conducted after excluding infants born at < 24 weeks of gestation. In this population, overall mortality for infants after live birth was 28.2% (267/948). The odds of mortality (aOR 0.93, 95% CI [0.88, 0.99]) and admission to the neonatal unit (0.93 [0.87, 0.98]) significantly decreased over time. Non-invasive ventilation support during stabilisation at birth increased significantly (1.26 [1.15, 1.38]) with corresponding decrease in mechanical ventilation at birth (0.89 [0.81, 0.97]) and following admission (0.80 [0.68–0.96]). Medical treatment for patent ductus arteriosus significantly decreased over the study period (0.90 [0.85, 0.96]). The incidence of major neonatal morbidities remained stable, except for a reduction in late-onset sepsis (0.94 [0.89, 0.99]). Gestation and centre of birth were significant independent factors for several outcomes. The results from our sensitivity analysis were compatible with our main results with the notable exception of death after admission to NICU (0.95 [0.89, 1.01]). There were significant improvements in survival and reduction of late-onset sepsis of extreme preterm infants in South Wales between 2007 and 2016. The sensitivity analysis suggests that some of the temporal changes observed were driven by improved outcomes in the most preterm of infants. Clinical practices related to respiratory support have changed but significant variations in clinical practices and outcomes between centres remain unexplained. The adoption of regional evidence-based clinical guidelines is likely to improve outcomes and reduce variation.

## Introduction

Although extreme preterm infants born at a gestational age of < 28 weeks are a small proportion of all births^1,2^ globally and in the UK, they contribute disproportionately to the burden of neonatal morbidity^1–3^, mortality^3,4^ and cost of intensive care^5^. Over the last few decades, survival rates of extremely preterm infants have steadily increased^2,3,6^ due to improvements in antenatal care and neonatal intensive care management. Despite improved survival, the incidence of morbidities unique to this population has not shown statistically significant reduction over the last decades^2,3,6^.

Medical teams taking care of extremely preterm infants need up-to-date outcome data to be able to counsel families and aid decision making^7^. Trend analyses of outcomes linked to clinical practices are crucial in identifying interventions that can lead to improved outcomes. Such analysis can also help to direct appropriate resources to support and promote specific areas of clinical practice, perpetuating a culture of continuous improvement and driving the establishment of research networks and quality improvement programmes worldwide^8^.

Compared to other European countries, the UK has higher rates of extreme and very preterm births^9,10^. Data from England and Wales show that 7% of all live births are preterm (born at < 37 completed weeks of gestation; 70 per 1000 live births). Of this 11% are very preterm (≥ 28 to < 32 weeks of gestation; 8 per 1000 live births) and 5% are extreme preterm (born at < 28 weeks of gestation; 4 per 1000 live births)^11^. Extreme preterm infants are at higher risk of severe morbidities and mortality^2,3^.

The three tertiary neonatal units in South Wales have been caring for extreme preterm infants for decades, and clinical care practices have undergone major changes over time. A comprehensive analysis of outcomes to guide local counselling of parents and to identify areas of improvement have not been conducted. We hypothesised that mortality, major morbidities, and care practices in this vulnerable group of infants would show statistically significant improvement over time, as reported in the literature. The objective of this study was to report the temporal trends of key outcomes (as described in the “Methods” section) and changes in respiratory management over 10 years in extremely preterm infants cared for in the South Wales Neonatal Network.

## Methods

### Setting

South Wales is home to 75% of the population of Wales, with England as its eastern border and mid-Wales forming the northern border. The estimated birth rate in South Wales is around 25,000 per year^11^ (https://statswales.gov.wales/Catalogue/Health-and-Social-Care/Births-Deaths-and-Conceptions). This neonatal care for the population is centralised and served by three tertiary neonatal intensive care units (NICUs) that offer a full range of medical care for preterm infants; one of these units also serves as a regional centre for neonatal surgery and other subspecialty services. Extreme preterm infants needing critical care were either born at one of the tertiary centres or transferred into these centres soon after birth. Besides, six local neonatal units provided limited high-dependency and special care for the more mature preterm infants closer to home either from birth or following repatriation from the tertiary units.

### Study population

The study cohort included all infants born alive at a gestational age of < 28 weeks between 1st January 2007 and 31st December 2016 at one of the three tertiary neonatal units in South Wales (inborn infants). This included live born infants who died in the delivery room following unsuccessful resuscitation. Infants born at < 28 weeks at any of the local neonatal units and transferred into one of the three tertiary units were also included in the cohort (outborn infants). No exclusion criteria were applied; however, extreme preterm infants who were born alive at local neonatal units but died before transfer to a tertiary unit were not included in the cohort. As a variable known to affect outcomes, statistical analysis accounted for the differences in the place of birth^12,13^.

### Data collection

From 2007, all three tertiary neonatal units contribute data for benchmarking purposes to the Vermont Oxford Network (VON) for all infants born at < 30 weeks of gestation or with birth weight < 1500 g. Data for the VON database was collected and verified by a designated senior clinician in each unit, ensuring consistency and high-quality. This study included prospectively collected anonymised data from all three tertiary neonatal units over a 10-year study period for infants < 28 weeks of gestation at birth.

Raw data included maternal and neonatal characteristics, resuscitation practices, early clinical management, and clinical outcomes including death and major morbidities. The data was collected for each infant for their first admission to the NICU until death or transfer to another centre or discharge home from the tertiary unit. Standard VON definitions were used for major morbidities (https://help.vtoxford.org/nightingale/help/#4662.htm) and are described in detail in Supplementary Table S1.

### Exposure variable, other variables, and outcomes

The primary exposure variable was the year of birth which was treated as a continuous variable in all statistical analysis.

The following unmodifiable co-variates were used for adjusted analyses:

1. Gestation (continuous variable).
2. Sex (binary variable).
3. Place of birth, i.e. inborn or outborn (binary variable).
4. Hospital of care (categorical variable with each hospital treated as a single category—three in total).

The co-primary outcomes included the following major outcomes:

1. Mortality after live birth and after admission to NICU.
2. Severe cranial ultrasound (CUSS) abnormalities, defined as a composite of severe intraventricular haemorrhage (IVH) grade III–IV^14^ OR periventricular leukomalacia.
3. Surgery for retinopathy of prematurity (ROP).
4. Bronchopulmonary dysplasia (BPD) at 36 weeks, defined as a requirement for mechanical ventilation, non-invasive ventilation, or supplemental oxygen at 36-weeks post-menstrual age.
5. Medical treatment for a patent ductus arteriosus (PDA) with indomethacin or ibuprofen.
6. Necrotising enterocolitis (NEC), defined as Bell stage II–III^15^.
7. Sepsis, defined as a culture-positive episode with bacterial or fungal organisms.

As death was a competing variable for all the major morbidity outcomes, a composite of death with each of the major morbidities was also analysed.

Secondary outcomes included key changes in respiratory practice including the use of surfactant, use of non-invasive respiratory support and use of mechanical ventilation.

### Statistical analysis

Statistical analysis was undertaken in SPSS version 25 (IBM Corporation, New York, USA). Missing or unavailable data were appropriately coded and excluded from analysis at all stages. These are presented in Supplementary Table S8. All data were analysed on a per-infant basis and not corrected for multiple gestations as these were not identifiable. Continuous data (gestation, birthweight, and birth head-circumference) are presented as medians with inter-quartile range (IQR) while categorical data are presented as percentages.

Trends were analysed by deriving multivariable logistic regression models in blocks with the outcome-of-interest (co-primary and secondary outcomes, as stated above) as the dependent variable.

1. In the first block, the dependent variable was modelled against the year of birth as an independent (continuous) variable and presented as unadjusted odds ratio (OR).
2. In the second block, the regression analyses were adjusted for confounders by including gestation at birth (continuous variable used as a decimal term for completed weeks and days), gender^16^ (binary variable coded as female = 0, male = 1), the centre of care^12^ (categorical variable coded as dummy variables) and outborn status^13,17^ (categorical variable coded as inborn = 0, outborn = 1) and presented as adjusted OR. Individual centres were not compared with each other; summary results testing centre as a factor are presented if reached statistical significance.
3. The third block was used to derive the most parsimonious model for each major outcome. All independent variables (year of birth, gestation, sex, place of birth and hospital of care) from block 2 were included in the model along with the clinically relevant interaction variables gestation at birth*year of birth, gestation at birth*gender and centre of care*outborn-status. A stepwise variable selection procedure (forward selection) was specified in which variables were sequentially entered into the model. The first variable considered for entry into the equation was the one with the largest positive or negative correlation with the dependent variable (outcome). This variable was entered into the equation only if it satisfied the criterion for entry (p-value < 0.05). If the first variable was entered, the independent variable not in the equation that had the largest partial correlation was considered next. The procedure stopped when there were no variables that met the entry criterion.

Due to the possibility of significant variation in the clinical care of infants born < 24 weeks of gestation, a sensitivity analysis of the co-primary and secondary outcomes was undertaken only for infants with a birth gestation between 24 and 27 weeks using the same methods as above.

A p-value of < 0.05 was considered statistically significant. All uses of the word “significant” in the results section refer to statistical significance only.

### Ethics

As this analysis was conducted on non-identifiable information, which was previously collected routinely for clinical purposes, it was excluded from specific ethical approval as per general guidance on the NHS Health Research Authority *guidance tool* for ethics approval. There was also no direct involvement of patients or their parents/guardians as none were identifiable. A data-sharing agreement was signed and approved by the NHS Research and Development Department at each site. Categorical variables with n < 5 have been suppressed in tables to prevent accidental identification of individuals and sites.

## Results

### Study population characteristics

In the 10 years, 948 infants born before 28 weeks of gestation were cared for in one of the three tertiary NICUs in South Wales; 741 (78.2%) were inborn. Median gestation of this population was 26.3 weeks (IQR 2.3 weeks). Of them, 100 (10.6%) were born between 22^+0^ and 23^+6^ weeks, 314 (33.1%) between 24^+0^ and 25^+6^ weeks and 534 (56.3%) between 26^+0^ and 27^+6^ weeks. The birth weight ranged from 310 to 1500 g (median 830 gm, IQR 321 gm) and the head circumference at birth (812 available) ranged from 18 to 37.4 cm (median 23.5 cm, IQR 3.0 cm). A slight male preponderance was recorded i.e. 54.7% (519/948; p = 0.63). Almost a quarter of the infants (23.3%; 221/948; p = 0.01) were multiple births, but the number of multiple pregnancies decreased over the study period (aOR 0.92 [95% CI 0.87, 0.98]). A major birth defect was reported in 5.3% (50/948) of the study population and this trend did not change significantly over time (p = 0.19). Detailed results on infant characteristics, maternal characteristics, resuscitation at delivery and respiratory support of infants after resuscitation are presented in Table 1 and Supplementary Tables S2–S5.

**Table 1:** Baseline demographics of the population (infant and maternal) by year.

Birth year (n)	2007	2008	2009	2010	2011	2012	2013	2014	2015	2016	p-value for trend
**Baseline characteristics**											
Infant											
Total infants	105	96	101	95	94	101	111	87	97	61	
Out-born	22	20	19	21	14	23	32	23	21	12	0.34
Birth gestation (weeks), median	26.7	26.1	26.1	26.1	26.3	26.1	26.0	26.0	26.0	26.6	
Birth weight (g), median	930	800	770	765	830	850	802.5	850	752	890	
Head circumference at birth (cm), median	24.5	23.4	23.0	23.1	23.3	23.8	23.5	23.5	23.0	23.8	
Male	59	52	61	50	52	49	64	48	49	35	0.63
Multiple birth	36	24	22	26	16	27	20	24	21	5	0.01
Major birth defect	£	5	11	5	5	10	5	£	£	£	0.19
Maternal											
Non-White	9	5	5	8	14	13	£	11	13	^£^	0.16
Prenatal care	104	92	100	95	92	95	109	86	96	58	0.56
Chorioamnionitis (from 2008)		19	7	21	24	21	15	15	18	6	0.71
Maternal hypertension, chronic or pregnancy-induced (from 2008)		11	23	12	9	14	14	10	7	8	0.10
Antenatal steroids exposure	91	74	83	76	80	91	91	78	82	52	0.12
Vaginal delivery	63	66	56	64	60	60	64	53	58	30	0.14
Antenatal magnesium sulphate (from 2012)						^£^	^£^	11	8	7	0.00

^£^Values < 5 suppressed to prevent accidental identification.

### Mortality (Table 2 and Supplementary Table S7)

**Table 2:** Key outcomes.

Key outcomes	10-year Incidence n (%age of available data)^$^	Available data n (%age)	Missing data n (%age)	Unadjusted odds (95% CI)	Adjusted odds (95% CI)^!^
Total infants	948				
Delivery room deaths	50 (5.3)	948 (0.0)	0 (0.0)	0.94 (0.85, 1.04)	1.01 (0.89, 1.15)^1,3^
Died after admission and before discharge	217 (22.9)	898 (0.0)	0 (0.0)	0.94 (0.89, 0.99)*	0.93 (0.87, .98)^1,2,6,^*
Any death after live birth	267 (28.2)	948 (0.0)	0 (0.0)	0.94 (0.89, 0.99)*	0.93 (0.88, 0.99)^1,2,3,4,6,7,^*
Severe IVH OR PVL (severe CUSS abnormalities)	151 (17.2)	876 (92.4)	72 (7.6)	0.97 (0.91, 1.03)	0.97 (0.91, 1.03)^1,2,3,4^
ROP surgery	94 (10.5)	944 (99.6)	4 (0.4)	0.96 (0.89, 1.03)	0.98 (0.91, 1.07)^1,3^
Bronchopulmonary dysplasia	290 (72.3)	401 (42.3)	547 (57.7)	1.00 (0.93, 1.09)	1.00 (0.92, 1.08)^1^
Medical treatment for PDA	239 (26.6)	898 (94.7)	50 (5.3)	0.93 (0.88, 0.98)*	0.90 (0.85, 0.96)^1,3^,*
Necrotising enterocolitis (NEC)	106 (11.8)	898 (94.7)	50 (5.3)	0.96 (0.89, 1.03)	0.96 (0.89, 1.04)^1,3,4,^
Any sepsis during stay	340 (37.9)	898 (94.7)	50 (5.3)	0.97 (0.93, 1.02)	0.98 (0.93, 1.03)^1,2,6,7^
Any early-onset sepsis	33 (3.7)	897 (94.6)	51 (5.4)	1.05 (0.93, 1.19)	1.05 (0.93, 1.20)
Any late-onset sepsis	317 (38.1)	831 (87.7)	117 (12.3)	0.96 (0.91, 1.00)	0.94 (0.89, 0.99)^1,2,7,^*

Odds (95% CI) are for trend, calculated by logistic regression models with the year of birth as a continuous variable, and adjusted for gestational age at birth^1^, sex^2^, centre^3^ and outborn-status^4^, along with the interaction terms gestation*year of birth^5^, gestation*gender (1)^6^ and centre of care*outborn-status (1)^7^.

^$^All analysis was conducted after excluding missing data.

^!^Superscript numbers indicate significant independent variable on adjusted analysis as numbered above.

**Figure 1:**
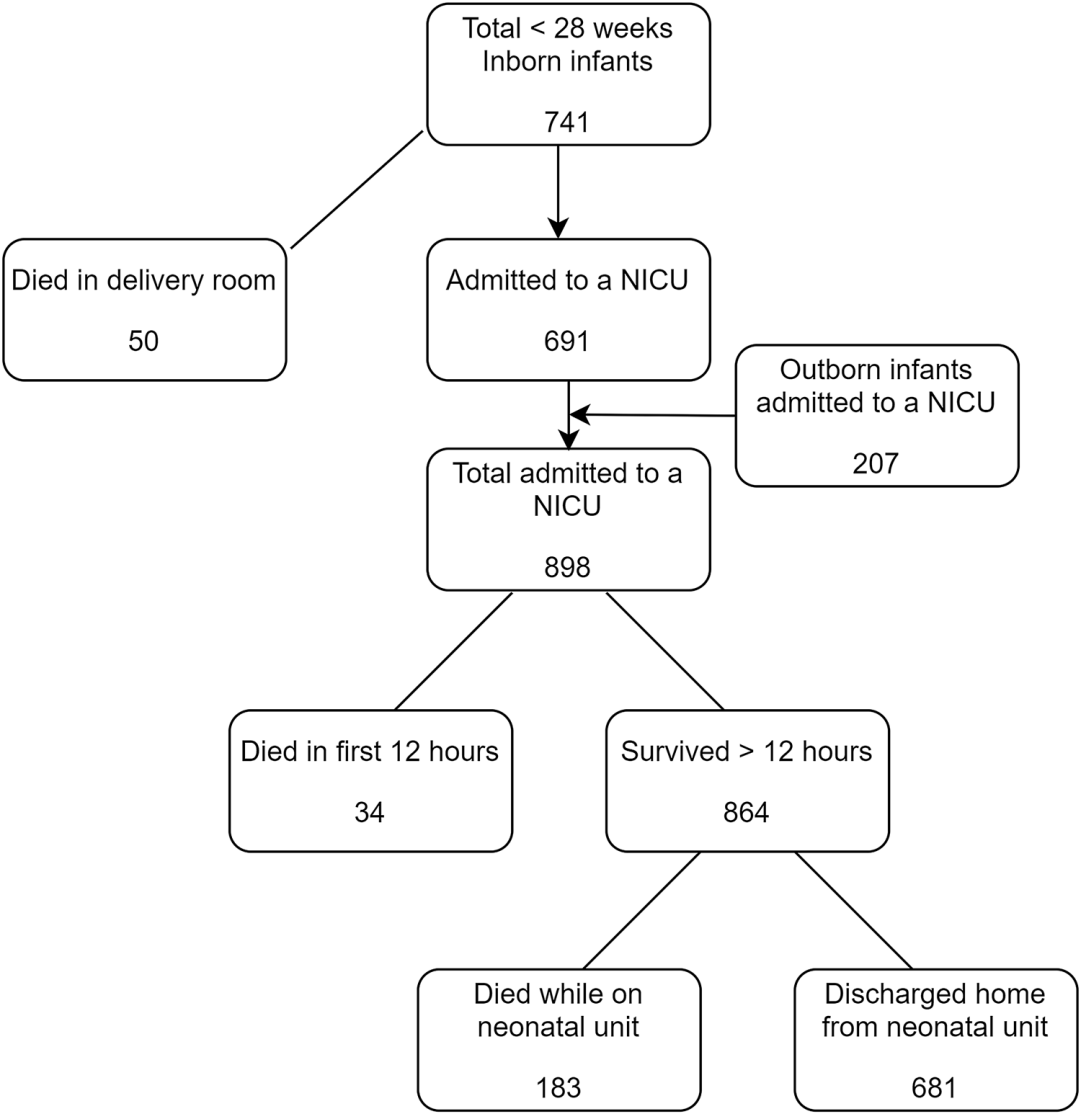
Summary of outcomes of extremely preterm infants after live birth between 2007 and 2016.

Figure 1 summarises the outcomes of all live births during the study period; summary of mortality outcomes is presented in Table 2. Of the 948 live born infants, 50 (5.3%) died in the delivery room. Of the 898 infants who were admitted to a NICU, 217 (24.2% of all admissions and 22.9% of all live births) died before discharge. There was a significant reduction in the odds of death after admission to NICU over the study period (aOR 0.93 [0.87, 0.98]), as was with increasing gestation at birth (aOR 0.65 [0.54, 0.78]). Overall mortality in this extreme preterm population after live birth and before discharge was 28.2% (267/948). The overall odds of mortality after live birth significantly reduced over the study period (aOR 0.93 [0.88, 0.99]) and with increasing gestation (aOR 0.60 [0.50, 0.71]). Boys had significantly higher odds of death after live birth (aOR 1.46 [1.06, 2.01]) and after admission to a unit (aOR 1.40 [1.00, 1.95]), although their odds of death reduced significantly with increasing gestation (p < 0.01) as in the full cohort. Centre of care was a significant independent variable for deaths (p < 0.01), indicating differences in outcome between centres. Detailed results are presented in Supplementary Table S7.

### Morbidities (Tables 2, 3 and Supplementary Table S6)

**Table 3:** Composite outcomes.

Key outcomes	10-year incidence n (%age of available data)^$^	Available data n (%age)	Missing data n (%age)	Unadjusted odds (95% CI)	Adjusted odds (95% CI)^!^
Death OR severe CUSS abnormalities	356 (37.8)	943 (99.5)	5 (0.5)	0.95 (0.90, 0.99)*	0.94 (0.90, 0.99)^1,2,3,^*
Death OR ROP surgery	356 (37.7)	944 (99.6)	4 (0.4)	0.94 (0.89, 0.98)*	0.94 (0.88, 0.99)^1,3,4,7^
Death or BPD	541 (82.1)	659 (69.5)	289 (30.5)	0.96 (0.89, 1.03)	0.95 (0.89, 1.03)^1^
Death or NEC	328 (34.6)	948 (0.0)	0 (0.0)	0.95 (0.91, 1.00)*	0.94 (0.89, 1.00)^1,2,3,6,^*
Death or sepsis during stay	536 (56.5)	948 (0.0)	0 (0.0)	0.94 (0.90, 0.98)*	0.93 (0.88, 0.98)^1,3,4,^*

Odds (95% CI) are for trend, calculated by logistic regression models with the year of birth as a continuous variable, and adjusted for gestational age at birth^1^, sex^2^, centre^3^ and outborn-status^4^, along with the interaction terms gestation*year of birth^5^, gestation*gender (1)^6^ and centre of care*outborn-status (1)^7^.

^$^All analysis was conducted after excluding missing data.

^!^Superscript numbers indicate significant independent variable on adjusted analysis as numbered above.

#### Neurological

Of the 898 infants admitted to a neonatal unit, 875 (97.4%) had a cranial ultrasound scan (CUSS) during their stay. Of these scans, 137 (15.7%) reported a severe intraventricular haemorrhage (IVH). Periventricular leukomalacia (PVL) was an uncommon finding (2.3%; 20/875) and did not change significantly over time (p = 0.08). The composite outcome of severe CUSS changes (severe IVH OR PVL) was noted in 151 infants (17.2%) decreased significantly with increasing gestation (aOR 0.73 [0.64, 0.84]) but did not change over time (aOR 0.97 [0.91, 1.03]. Outborn infants who were transferred into the tertiary units after birth had higher odds of developing severe CUSS changes (aOR 1.66 [1.10, 2.48]). The composite of death OR severe CUSS changes decreased with increasing gestation (aOR 0.55 [0.49, 0.61]) and over the study period (aOR 0.94 [0.90, 0.99]). The proportion of missing data was small, primarily contributed by infants who died in the delivery room or within 12-h who did not have a CUSS performed.

Incidence of surgery for ROP remained stable over the study period (aOR 0.98 [0.91, 1.07]), although the composite of death OR ROP surgery reduced marginally (aOR 0.94 [0.88, 0.99]). Odds for both outcomes reduced with increasing gestation (p < 0.01 for both), and there was a significant centre effect (p < 0.01 for both).

#### Respiratory

Of the 401 infants for whom data was available, 290 infants (72.3% of available data) developed bronchopulmonary dysplasia (BPD) after live birth. The incidence of BPD did not change significantly over the study period (aOR 1.00 [0.92, 1.08]) but the odds of developing BPD was significantly lower at the higher gestational ages (aOR 0.69 [0.57, 0.84]). Accounting for deaths, the odds of the composite outcome death OR BPD (541/659; 82.1%) did not change over the study period (aOR 0.95 [0.89, 1.03]), although this decreased with increasing gestation (aOR 0.60 [0.50, 0.70]). However, there was a significant proportion of missing data (57.7% for BPD and 30.5% for death OR BPD).

#### Cardiovascular

The odds of medical treatment for diagnosed PDA with either indomethacin or ibuprofen decreased significantly over time (aOR 0.90 [0.85, 0.68]) and was less likely with increasing gestation (aOR 0.70 [0.62, 0.79]).

#### Gastro-intestinal

Of the 898 infants who were admitted to a neonatal unit, 106 (11.8%) were reported to have necrotising enterocolitis (NEC) and 63 (7%) had surgery for NEC, suspected NEC, or bowel perforation. The odds of NEC were stable over the study period (aOR 0.96 [0.89, 1.04]) but significantly decreased with increasing gestational age (aOR 0.78 [0.67, 0.91]). Outborn infants had significantly higher odds for developing NEC during the study period (aOR 5.04 [2.84, 8.94]). The centre of care had a significant effect on this outcome (p < 0.01), most likely due to cases being concentrated in one surgical centre.

#### Infections

During the study period, 340 infants developed sepsis; of these, 33 were early-onset (before 72-h of life) and the rest were late-onset (beyond 72-h of life). The odds of late-onset sepsis (LOS) reduced over time (aOR0.94 [0.89, 0.99]), with increasing gestation (aOR 0.68 [0.61, 0.77]) and in boys (aOR 0.66 [0.49, 0.89]). Missing data for sepsis was due to the early death of infants and the data was complete for the composite of death OR sepsis.

### Changes in respiratory practice (Table 4 and Supplementary Tables S4, S5)

**Table 4:** Key changes in the practice of respiratory support.

Key changes in practice	10-year incidence n (%age of available data)^$^	Available data n (%age)	Missing data n (%age)	Unadjusted odds (95% CI)	Adjusted odds (95% CI)^!^
Total infants	948				
Surfactant during initial resuscitation	855 (90.2)	948 (0.0)	0 (0.0)	0.94 (0.87, 1.01)	0.93 (0.86, 1.01)^1,3,7^
Surfactant at any time (absolute)	894 (94.3)	948 (0.0)	0 (0.0)	0.93 (0.84, 1.03)	0.90 (0.81, 1.00)^1,3,4,^*
Any non-invasive ventilation during resuscitation (face-mask, CPAP)	842 (90.6)	929 (98.0)	19 (2.0)	1.28 (1.17, 1.39)*	1.26 (1.15, 1.38)^3,7,^*
Any non-invasive ventilation after resuscitation	733 (81.6)	898 (94.7)	50 (5.3)	1.03 (0.97, 1.09)	1.03 (0.97, 1.10)^1,2^
Any mechanical ventilation during resuscitation	871 (92)	947 (99.9)	1 (0.1)	0.90 (0.82, 0.98)*	0.89 (0.81, 0.97)^1,3,7,^*
Any mechanical ventilation after initial resuscitation	878 (97.8)	898 (94.7)	50 (5.3)	0.80 (0.67, 0.95)*	0.80 (0.68, 0.96)^1,^*

Odds (95% CI) are for trend, calculated by logistic regression models with the year of birth as a continuous variable, and adjusted for gestational age at birth^1^, sex^2^, centre^3^ and outborn-status^4^, along with the interaction terms gestation*year of birth^5^, gestation*gender (1)^6^ and centre of care*outborn-status (1)^7^.

^$^All analysis was conducted after excluding missing data.

^!^Superscript numbers indicate significant independent variable on adjusted analysis as numbered above.

The odds of using non-invasive respiratory support during resuscitation significantly increased (aOR 1.26 [1.15, 1.38]) while mechanical ventilation during (aOR 0.89 [0.81, 0.97]) or after (aOR 0.80 [0.68, 0.96]) resuscitation significantly decreased over the study period. Use of surfactant decreased marginally over the study period (aOR 0.90 [0.81, 1.00]). All these outcomes were dependent on gestation, with the use of non-invasive ventilation increasing with gestation (p < 0.01) and mechanical ventilation decreasing with gestation (p < 0.01).

### Sensitivity analysis

After excluding the 100 infants who were born at < 24 weeks of gestation, a sensitivity analysis was conducted for the co-primary and secondary outcomes. Point estimates (Supplementary Tables S9, S10) were compatible with main analysis, although death after admission to NICU (aOR 0.95 [0.89, 1.01]) and the composite death OR ROP Surgery (aOR 0.95 [0.89, 1.00]) may have attenuated (although overall mortality remained lower). For clinical care, there was a significant reduction in the use of surfactant during initial resuscitation (aOR 0.90 [0.82, 0.99]) but the use of surfactant at any time after live birth failed to reach statistical significance (aOR 0.87 [0.76, 1.00]).

## Discussion

### Summary

We have presented longitudinal outcomes of a large population of extremely preterm infants born and cared for in the tertiary NICUs in South Wales. To our knowledge, this is the first detailed multi-centre trend analysis of mortality, morbidity, and clinical practices of a large defined extreme preterm population in Wales.

### Discussion of main results

The overall mortality before transfer or discharge was 28.2% for all live births in the population with a significant reduction in odds of mortality after admission to NICU^3,6,18^. As expected, the odds of mortality after birth and after admission to NICU were inversely proportional to gestational age at birth. Our mortality figures after live birth were lower than other comparable populations from the UK^6^ and France^19^, but similar to a large US population^3^.

In keeping with current evidence^20^ and practice^21^, non-invasive respiratory support of extremely preterm infants at birth steadily increased during the study period (19%) with a corresponding decline in the odds of endotracheal intubation (14%). More mature infants had higher odds of being intubated at birth, possibly secondary to the emerging use of INSURE technique to administer early surfactant to facilitate rapid extubation and before the increasing popularity of using LISA techniques. Mechanical ventilation on conventional modes at any time during stay significantly reduced over the study period vindicating our hypothesis that infants were spending less time on mechanical ventilation at any time over the study period, although there was a modest but significant increase in the use of high-frequency ventilation. Use of the most recent mode of neonatal respiratory support—high flow nasal cannula therapy—significantly increased over the study period^21^.

Cohort studies on extreme preterm gestation suggest that the approach to infants < 24 weeks of gestation is variable; some of them may not receive active resuscitation or care^22^. The results from our sensitivity analysis, after excluding infants < 24 weeks of gestation, were compatible with our main results with the notable exception of death after admission to NICU. This possibly suggests that some of the temporal changes in mortality which were observed in the whole cohort may have been driven by the improved outcome in the most preterm infants born at < 24 weeks of gestation.

The incidence of the major neonatal morbidities (BPD, PDA, NEC, IVH, PVL, ROP) remained largely static over the study period except for LOS. Although the decreasing trend was reflective of the overall UK trend, the incidence of LOS reported in our population was higher than published UK data^23^ but half that of a comparable population in the US^3^. Similarly, severe IVH and severe ROP was higher in our population than in other international and European studies^6,8,19^. Although the reasons for these differences in outcomes are unclear, it could be related to better survival in our population. We found a higher incidence of IVH but a lower incidence of LOS in boys compared to girls; this contrasts with the pattern reported before and warrants further research. Although the treatment for PDA remains subjective, our results are in keeping with reported trends from the USA^24^. It is important to point out that the incidence of BPD has reportedly been on the rise in other comparable populations from the USA^3^ and UK^8,18^. While our stable trend for most neonatal morbidities may be reassuring to some, one may argue that the lack of improvement may reflect the lag in translating improving practices from research studies^20^ into clinical practice^19^. This slow pace is not uncommon in trend analysis of population outcome^3,6,8^.

We reported significant variations in multiple areas of clinical practice as well as outcomes between participating centres. These included practices related to resuscitation, respiratory support, and management of PDA as well as outcomes such as IVH, ROP and sepsis. Differences in rates of surgery could be explained by the fact that only one of the centres offered neonatal surgical care. While variation between centres is well known in neonatal practice^12,25^, exploring the cause for this difference was beyond the scope of this paper and likely to be multifactorial. The adoption of evidence-based network and national guidelines should significantly reduce some of the variations observed but must be balanced with the need for tailoring individualised care and clinician’s autonomy for wise professional judgment.

### Missing data

Like other retrospective studies from clinical databases, we had to account for missing data in several data fields (Supplementary Table S8). For the main outcome measures, the proportion of missing data varied from 5 to 7%. This was primarily because of early death where the recording of such outcome measures was not plausible. For mortality itself, and the composite outcomes incorporating mortality, our study had negligible missing data (< 1%). The notable exception was BPD, where the outcome reached the definition threshold towards the latter end of the neonatal stay. This meant many outborn infants were transferred to a non-participating special care unit for continuing care closer to the family’s home as part of operations guideline of a managed clinical network. Consequently, while other outcomes in this work have good data completeness the outcomes for BPD need to be interpreted with caution.

### Limitations

Our study has several limitations. While the large majority of the extreme preterm infants born alive in South Wales were included in our analysis, infants who died in the delivery room at local neonatal units before transfer to the tertiary units could not be captured in this data set. Although our study spanned 10 years, the number of infants in each gestation week was still insufficient to undertake a meaningful subgroup analysis. We have adjusted for known confounders, but other unmeasured factors may also have had an impact on the outcomes presented. It was beyond the scope of our study to explore the link between care practices and outcomes; this is planned for a later stage. We have used ‘centre’ as a simple adjusting variable which hides further complexity including differences in care practices, the intensity of work^26^, and local population differences.

### Strengths

However, our study has several strengths which need highlighting. We have followed recommendations for reporting outcomes of extreme preterm infants^7,27^. We have restricted our analysis to the extreme preterm population who are at the highest risk for morbidity and mortality after birth. Our population was well defined due to the model of centralised neonatal care as part of a managed clinical network in South Wales. The inception point for outcomes was ‘live birth’ which effectively included all infants born with signs of life, thus reducing selection bias. The outcome definitions are embedded in the internationally accepted VON criteria, and additional outcomes have been defined clearly before analysis including the timing of assessment where relevant. Information bias was likely to be minimal considering that data input into the VON database was undertaken in each unit by senior clinicians with substantial experience and VON data collection and interpretation of outcome definitions. Our analysis is detailed, longitudinal, adjusted for common confounders, and reports uncertainty ranges for all outcomes.

Extreme preterm infants in South Wales remain vulnerable to multiple major morbidities and mortality after birth. While opportunities for improvement in care and outcomes remain and are the topics of current research studies, major changes can only be expected from a significant reduction in the preterm population being born at this extreme range of gestation. Currently, no effective treatment options are available to significantly delay or prevent premature birth^28^. Research into more accurate prediction^29,30^ and prevention^28^ of preterm delivery should become the focus of healthcare and research funding bodies worldwide to enable further improvements in outcomes of preterm infants.

In summary, we have reported detailed outcomes of an extreme preterm population who were born alive over 10 years in Wales. This is the first such report from Wales, and our data will help counsel parents and plan further improvements in South Wales.

## Supplementary information

Supplementary Information.

## Data Availability

The corresponding author had full access to all the data in the study and had final responsibility for the decision to submit for publication.

## Abbreviations

aOR: Adjusted odds ratio
BPD: Bronchopulmonary dysplasia
CUSS: Cranial ultrasound scan
IQR: Inter-quartile range
IVH: Intraventricular haemorrhage
LOS: Late-onset sepsis
NEC: Necrotising enterocolitis
NICU: Neonatal intensive care unit
OR: Odds ratio
PDA: Patent ductus arteriosus
PVL: Periventricular leukomalacia
ROP: Retinopathy of prematurity
VON: Vermont Oxford network
95% CI: 95% Confidence interval
