# Supplementary material for: Temporal trends of care practices, morbidity, and mortality of extremely preterm infants over 10-years in South Wales, UK
Source: Sci Rep. 2020 Oct 30;10:18738. doi: 10.1038/s41598-020-75749-4 (PMC7603316; doi:10.1038/s41598-020-75749-4)
Supplement: Supplementary file 1 — Supplementary Information. [file 41598_2020_75749_MOESM1_ESM.docx]

**Temporal trends of care practices, morbidity and mortality of extremely preterm infants till discharge between 2007-2016 in South Wales, UK**

Lieve Boel MD ^1^, Megan Clark MBChB ^2^, Annabel Greenwood MRCPCH ^1^, Alok Sharma MD ^1^, Sujoy Banerjee MD ^3^, Nitin Goel MD ^1^, Gautam Bagga MRCPCH ^4^, Chuen Poon PhD ^4^, David Odd MD ^5^, Mallinath Chakraborty PhD ^1,6^

^1^ Regional Neonatal Intensive Care Unit, University Hospital of Wales, Cardiff, UK

^2^ School of Medicine, Cardiff University, Cardiff, UK

^3^ Neonatal Intensive Care Unit, Singleton Hospital, Swansea, UK

^4^ Neonatal Intensive Care Unit, Royal Gwent Hospital, Newport, UK

^5^ Division of Population Medicine, School of Medicine, Cardiff University, Cardiff, UK

^6^ Centre for Medical Education, School of Medicine, Cardiff University, Cardiff, UK

**Supplementary Information**

| **Morbidity** | **Definition** |
| --- | --- |
| bronchopulmonary dysplasia (BPD) | need for supplemental oxygen, conventional ventilation or non-invasive ventilation at 36 weeks post-menstrual age (PMA)^1^ |
| necrotising enterocolitis (NEC) | stage II or III as defined by the modified Bell staging criteria^2^ |
| severe intra-ventricular haemorrhage (IVH) | grade three or four in the Papile classification for IVH^3^ |
| retinopathy of prematurity (ROP) | defined by the international classification of ROP^4^ |
| severe ROP | stage three or higher on this classification scale^4^ |
| Early-onset sepsis (EOS) | a bacterial pathogen cultured on blood or cerebrospinal fluid before the third day of life |
| Late-onset sepsis (LOS) | a bacterial pathogen cultured on blood or cerebrospinal fluid after the third day of life |

Supplementary Table 1: Definitions of major morbidities used in the study.

| **Infant Characteristics** | **Birth year (n)** | | | | | | | | | | **Unadjusted odds (95% CI) ^$^** | **Adjusted odds (95% CI) ^$ !^** |
| --- | --- | --- | --- | --- | --- | --- | --- | --- | --- | --- | --- | --- |
|  | **2007** | **2008** | **2009** | **2010** | **2011** | **2012** | **2013** | **2014** | **2015** | **2016** |  |  |
| Total infants | 105 | 96 | 101 | 95 | 94 | 101 | 111 | 87 | 97 | 61 |  |  |
| Birth Gestation (weeks), median | 26.7 | 26.1 | 26.1 | 26.1 | 26.3 | 26.1 | 26.0 | 26.0 | 26.0 | 26.6 |  |  |
| Birth weight (gram), median | 930 | 800 | 770 | 765 | 830 | 850 | 802.5 | 850 | 752 | 890 |  |  |
| Head circumference at birth (cm), median | 24.5 | 23.4 | 23.0 | 23.1 | 23.3 | 23.8 | 23.5 | 23.5 | 23.0 | 23.8 |  |  |
| Male | 59 | 52 | 61 | 50 | 52 | 49 | 64 | 48 | 49 | 35 | 0.99 (0.94, 1.04) | 0.99 (0.95, 1.04) |
| Multiple birth | 36 | 24 | 22 | 26 | 16 | 27 | 20 | 24 | 21 | 5 | 0.93 (0.88, 0.98) * | 0.93 (0.87, 0.98) *  ^1^ 0.88 (0.79, 0.98)  ^3^ p<0.01  ^4^ 0.66 (0.44, 0.98) |
| Out-born | 22 | 20 | 19 | 21 | 14 | 23 | 32 | 23 | 21 | 12 | 1.03 (0.97, 1.09) | 1.03 (0.97, 1.09)  ^1^ 1.21 (1.08, 1.36) * |
| Major birth defect | £ | 5 | 11 | 5 | 5 | 10 | 5 | £ | £ | £ | 0.93 (0.84, 1.04) | 0.96 (0.86, 1.07)  ^3^ p<0.01  ^4^ 0.39 (0.15, 0.99) |

Supplementary table 2: Characteristics of infants. Odds (95% CI) are for trend, calculated by logistic regression models with the year of birth as a continuous variable, and adjusted for gestational age at birth^1^, sex^2^, centre^3^, and outborn-status^4^, along with the interaction terms gestation*year of birth^5^, gestation*gender(1)^6^ and centre of care*outborn-status(1)^7^. ^$^ = missing data was excluded before analysis, ^£^ = values <5 suppressed to prevent accidental identification. ^!^ Superscript numbers indicate significant independent variable on adjusted analysis as numbered above.

Detailed maternal characteristics by birth year are presented in supplementary table 3. Maternal race or ethnicity was predominantly white (855/941; 90.9%); with the rest noted to have another ethnic background (missing data on 7 infants). Chorioamnionitis or maternal hypertension was present in 17.4% (146/839) and 12.8% (108/842) of the pregnancies, respectively. Although there were no changes in the temporal trend for either one of these complications, the odds of chorioamnionitis was lower and the odds of hypertension was higher in more mature infants. Vaginal delivery was less likely with increasing gestational age but outborn infants were more likely to have been born vaginally. Both prenatal care and antenatal steroid exposure were high in expectant mothers. Data about antenatal magnesium sulphate were available from 2012; it was used in only 6.2% (28/451) of the infants but the odds of its use increased significantly between 2012 and 2016 (aOR 1.795 [1.307, 2.465]).

| **Maternal characteristics** | **Birth year (n)** | | | | | | | | | | **Unadjusted odds (95% CI) ^$^** | **Adjusted odds (95% CI) ^$ !^** |
| --- | --- | --- | --- | --- | --- | --- | --- | --- | --- | --- | --- | --- |
|  | **2007** | **2008** | **2009** | **2010** | **2011** | **2012** | **2013** | **2014** | **2015** | **2016** |  |  |
| Total infants | 105 | 96 | 101 | 95 | 94 | 101 | 110 | 87 | 93 | 59 |  |  |
| Non-White | 9 | 5 | 5 | 8 | 14 | 13 | £ | 11 | 13 | £ | 1.06 (0.98, 1.15) | 1.09 (1.00, 1.18) *  ^3^ P<0.01 |
| Prenatal care | 104 | 92 | 100 | 95 | 92 | 95 | 109 | 86 | 96 | 58 | 0.95 (0.81, 1.12) | 0.96 (0.82, 1.13) |
| Chorioamnionitis (from 2008) |  | 19 | 7 | 21 | 24 | 21 | 15 | 15 | 18 | 6 | 0.99 (0.92, 1.06) | 1.01 (0.94, 1.09)  ^1^ 0.84 (0.74, 0.95)  ^3^ P<0.01  ^4^ 0.58 (0.35, 0.96) |
| Maternal hypertension, chronic or pregnancy-induced (from 2008) |  | 11 | 23 | 12 | 9 | 14 | 14 | 10 | 7 | 8 | 0.93 (0.86, 1.01) | 0.93 (0.85, 1.01)  ^1^ 1.66 (1.39, 2.00)  ^2^ 0.63 (0.41, 0.95) |
| Antenatal steroids exposure | 91 | 74 | 83 | 76 | 80 | 91 | 91 | 78 | 82 | 52 | 1.05 (0.99, 1.12) | 1.05 (0.98, 1.12)  ^1^ 1.49 (1.31, 1.70)  ^3^ p=0.04  ^4^ 0.47 (0.31, 0.71) |
| Vaginal Delivery | 63 | 66 | 56 | 64 | 60 | 60 | 64 | 53 | 58 | 30 | 0.97 (0.92, 1.01) | 0.95 (0.91, 1.00)  ^1^ 0.52 (0.46, 0.59)  ^4^ 1.60 (1.13, 2.25) |
| Antenatal Magnesium Sulphate (from 2012) |  |  |  |  |  | £ | £ | 11 | 8 | 7 | 1.80 (1.31, 2.47) * | 1.80 (1.31, 2.47) * |

Supplementary table 3: Maternal characteristics of the cohort. Odds (95% CI) are for trend, calculated by logistic regression models with the year of birth as a continuous variable, and adjusted for gestational age at birth^1^, sex^2^, centre^3^, and outborn-status^4^, along with the interaction terms gestation*year of birth^5^, gestation*gender(1)^6^ and centre of care*outborn-status(1)^7^. ^$^ = missing data was excluded before analysis, ^£^ = values <5 suppressed to prevent accidental identification. All maternal data were analysed on a per-infant basis as multiple pregnancies could not be identified. ^!^ Superscript numbers indicate significant independent variable on adjusted analysis as numbered above.

Care practices to support the infant’s breathing immediately after delivery have changed over the study period (supplementary table 4). The use of face-mask ventilation (75.7% in 2007 to 93.4% in 2016) and nasal continuous positive airway pressure (nCPAP, data for 2011-2016) significantly increased over the study period, although nCPAP was more often used in infants born at higher gestational ages and in boys. In parallel, the use of endotracheal tube (ETT) ventilation during resuscitation at birth significantly decreased from 96% in 2007 to 82% in 2016, although more mature infants were more likely to be intubated at birth. Use of oxygen (96.6%; 915/947, p = 0.70) and administration of surfactant (90.2%; 855/948; p = 0.09) during initial resuscitation remained unchanged over time but were more likely with increasing gestational age. Also, the centre of care seemed to have a significant effect on the use of non-invasive respiratory support, ETT ventilation and the administration of surfactant during initial resuscitation at birth. The use of chest compression or adrenaline during resuscitation at birth remained low at 10.5% (99/947; p = 0.27) and 3.4% (32/948; p = 0.45), respectively.

| **Resuscitation at delivery** | **Birth year (n)** | | | | | | | | | | **Unadjusted odds (95% CI) ^$^** | **Adjusted odds (95% CI) ^$ !^** |
| --- | --- | --- | --- | --- | --- | --- | --- | --- | --- | --- | --- | --- |
|  | **2007** | **2008** | **2009** | **2010** | **2011** | **2012** | **2013** | **2014** | **2015** | **2016** |  |  |
| Total infants | 105 | 96 | 101 | 95 | 94 | 101 | 110 | 87 | 93 | 59 |  |  |
| Oxygen during initial resuscitation | 103 | 90 | 96 | 93 | 94 | 98 | 108 | 84 | 91 | 58 | 0.98 (0.86, 1.11) | 0.97 (0.85, 1.10)  ^1^ 1.69 (1.30, 2.18) |
| Face mask ventilation during initial resuscitation  (including CPAP till 2010) | 78 | 74 | 83 | 90 | 87 | 90 | 107 | 81 | 87 | 57 | 1.24 (1.14, 1.34) * | 1.22 (1.12, 1.32) *  ^3^ p<0.01  ^4^ 3.67 (1.26, 10.71)  ^7^ p=0.02 |
| Nasal CPAP during initial resuscitation (from 2011, including nasal IMV and nasal SMV) |  |  |  |  | £ | 8 | 6 | 16 | 14 | 18 | 1.60 (1.34, 1.92) * | 1.66 (1.38, 2.01) *  ^1^ 2.26 (1.54, 3.32)  ^2^ 0.54 (0.31, 0.94)  ^6^ 0.54 (0.33, 0.89) |
| Any non-invasive ventilation during resuscitation (face-mask, CPAP) | 78 | 74 | 83 | 90 | 87 | 90 | 109 | 83 | 88 | 60 | 1.28 (1.17, 1.39) * | 1.26 (1.15, 1.38) *  ^3^ P < 0.01  ^7^ P=0.01 |
| Endotracheal tube ventilation during initial resuscitation | 101 | 90 | 90 | 90 | 86 | 96 | 105 | 76 | 87 | 50 | 0.90 (0.82, 0.98) * | 0.89 (0.81, 0.97) *  ^1^ 1.24 (1.05, 1.46)  ^3^ P<0.01  ^7^ P<0.01 |
| Surfactant during initial resuscitation | 99 | 86 | 87 | 87 | 86 | 97 | 102 | 77 | 85 | 49 | 0.94 (0.87, 1.01) | 0.93 (0.86, 1.01)  ^1^ 1.20 (1.03, 1.39)  ^3^ P<0.01  ^7^ P<0.01 |

Supplementary table 4: Resuscitation practices. Odds (95% CI) are for trend, calculated by logistic regression models with the year of birth as a continuous variable, and adjusted for gestational age at birth^1^, sex^2^, centre^3^, and outborn-status^4^, along with the interaction terms gestation*year of birth^5^, gestation*gender (1)^6^ and centre of care*outborn-status (1)^7^. $ = missing data was excluded before analysis, £ = values <5 suppressed to prevent accidental identification. ^!^ Superscript numbers indicate significant independent variable on adjusted analysis as numbered above.

The use of mechanical ventilation as a mode of respiratory support significantly reduced, both over time (from 98% (101/103) in 2007 to 90% (54/60) in 2016 and with increasing gestational age. High-frequency oscillatory ventilation (HFOV) was less commonly used (30%; 269/898) than conventional mechanical ventilation (97.8%; 878/898). Nasal CPAP was more likely to be used in infants born at higher gestational age but less likely in boys. 37.3% (335/898) of the neonates in the study population had respiratory support via high flow nasal cannula (HFNC) after initial resuscitation. The use of this mode of respiratory support has significantly increased over time (from 21.4% (22/103) in 2007 to 83.3% (50/60) in 2016) and with increasing gestational age, but these effects were significantly different between centres. Most infants received oxygen (99.8%; 896/898) and/or surfactant (99.5%; 894/898) during their stay on the neonatal unit. The use of both interventions did not change significantly over time, but the use of surfactant increased with increasing gestational age.

| **Respiratory support for infants** | **Birth year (n)** | | | | | | | | | | **Unadjusted odds (95% CI) ^$^** | **Adjusted odds (95% CI) ^$ !^** |
| --- | --- | --- | --- | --- | --- | --- | --- | --- | --- | --- | --- | --- |
|  | **2007** | **2008** | **2009** | **2010** | **2011** | **2012** | **2013** | **2014** | **2015** | **2016** |  |  |
| Total infants | 105 | 96 | 101 | 95 | 94 | 101 | 111 | 87 | 97 | 61 |  |  |
| Surfactant at any time during stay | 102 | 91 | 93 | 88 | 90 | 99 | 106 | 82 | 90 | 53 | 0.93 (0.84, 1.03) | 0.90 (0.801, 1.00) *  ^1^ 1.57 (1.30, 1.91)  ^3^ P<0.01  ^4^ 4.44 (1.36, 14.57) |
| Oxygen after initial resuscitation | 102 | 90 | 91 | 87 | 85 | 99 | 107 | 86 | 90 | 59 | 0.98 (0.60, 1.60) | 1.00 (0.61, 1.64) |
| Conventional tube ventilation after initial resuscitation | 101 | 90 | 89 | 87 | 83 | 98 | 105 | 82 | 89 | 54 | 0.80 (0.67, 0.95) * | 0.80 (0.68, 0.96) *  ^1^ 0.44 (0.26, 0.74) |
| High frequency ventilation after initial resuscitation | 28 | 23 | 29 | 13 | 28 | 29 | 41 | 28 | 37 | 13 | 1.05 (1.00, 1.11) | 1.07 (1.01, 1.13) *  ^1^ 0.57 (0.48, 0.68)  ^3^ P<0.01  ^6^ 1.27 (1.00, 1.60) |
| Any mechanical ventilation after initial resuscitation | 101 | 90 | 89 | 87 | 83 | 98 | 105 | 82 | 89 | 54 | 0.80 (0.67, 0.95) * | 0.80 (0.68, 0.96) *  ^1^ 0.44 (0.26, 0.74) |
| Nasal CPAP after initial resuscitation | 88 | 70 | 69 | 70 | 66 | 77 | 89 | 71 | 73 | 53 | 1.03 (0.97, 1.09) | 1.03 (0.97, 1.10)  ^1^ 2.10 (1.82, 2.42)  ^2^ 0.60 (0.42, 0.87) |
| Nasal IMV or nasal SIMV after initial resuscitation | 21 | 28 | 23 | 40 | 33 | 24 | 24 | 16 | 16 | 12 | 0.94 (0.89, 0.99) * | 1.02 (0.95, 1.09)  ^3^ P<0.01 |
| High flow nasal cannula after initial resuscitation (absolute) | 22 | £ | £ | 5 | 24 | 43 | 63 | 59 | 66 | 50 | 1.66 (1.55, 1.78) * | 1.70 (1.58, 1.83) *  ^1^ 1.39 (1.22, 1.59)  ^3^ P<0.01 |
| Any non-invasive ventilation after resuscitation | 90 | 70 | 69 | 70 | 66 | 77 | 91 | 72 | 75 | 53 | 1.03 (0.97, 1.10) | 1.04 (0.97, 1.11)  ^1^ 2.20 (1.90, 2.55)  ^2^ 0.61 (0.42, 0.89) |

Supplementary table 5: Respiratory support of infants after initial resuscitation. Odds (95% CI) are for trend, calculated by logistic regression models with the year of birth as a continuous variable, and adjusted for gestational age at birth^1^, sex^2^, centre^3^, and outborn-status^4^, along with the interaction terms gestation*year of birth^5^, gestation*gender (1)^6^ and centre of care*outborn-status (1)^7^. $ = missing data was excluded before analysis, £ = values <5 suppressed to prevent accidental identification. ^!^ Superscript numbers indicate significant independent variable on adjusted analysis as numbered above.

| **Details of Morbidity** | **Birth year (n)** | | | | | | | | | | **Unadjusted odds (95% CI) ^$^** | **Adjusted odds (95% CI) ^$ !^** |
| --- | --- | --- | --- | --- | --- | --- | --- | --- | --- | --- | --- | --- |
|  | **2007** | **2008** | **2009** | **2010** | **2011** | **2012** | **2013** | **2014** | **2015** | **2016** |  |  |
| Total infants | 105 | 96 | 101 | 95 | 94 | 101 | 111 | 87 | 97 | 61 |  |  |
| Any intraventricular haemorrhage (IVH) | 43 | 39 | 45 | 39 | 46 | 46 | 44 | 32 | 42 | 24 | 0.98 (0.93, 1.03) | 0.98 (0.93, 1.03)  ^1^ 0.69 (0.62, 0.77)  ^2^ 1.91 (1.43, 2.53)  ^3^ P<0.01  ^4^ 1.50 (1.08, 2.10) |
| Severe IVH (Grade 3-4) | 15 | 13 | 17 | 14 | 13 | 15 | 16 | 12 | 14 | 8 | 0.98 (0.92, 1.05) | 0.98 (0.92, 1.05)  ^1^ 0.70 (0.61, 0.81)  ^2^ 1.77 (1.20, 2.60)  ^3^ P=0.01  ^4^ 1.67 (1.09, 2.54) |
| Severe IVH OR Peri-ventricular leukomalacia (PVL) = Severe cranial ultrasound abnormalities (CUSS) | 18 | 16 | 18 | 14 | 13 | 19 | 17 | 13 | 15 | 8 | 0.97 (0.91, 1.03) | 0.97 (0.91, 1.03)  ^1^ 0.73 (0.64, 0.84)  ^2^ 1.65 (1.14, 2.38)  ^3^ P=0.02  ^4^ 1.66 (1.10, 2.48) |
| Death OR Severe CUSS | 37 | 42 | 46 | 38 | 39 | 40 | 36 | 27 | 35 | 16 | 0.95 (0.90, 0.99) * | 0.94 (0.90, 0.99) *  ^1^ 0.55 (0.49, 0.61)  ^2^ 1.54 (1.15, 2.06)  ^3^ P<0.01 |
| ROP stage ≥ 3 | 17 | 18 | 15 | 15 | 10 | 11 | 19 | 15 | 17 | 8 | 0.94 (0.88, 1.01) | 0.96 (0.89, 1.04)  ^1^ 0.53 (0.45, 0.63)  ^3^ P<0.01 |
| ROP surgery | 14 | 10 | 13 | 10 | £ | 7 | 12 | 10 | 10 | £ | 0.96 (0.89, 1.03) | 0.98 (0.91, 1.07)  ^1^ 0.62 (0.53, 0.74)  ^3^ P<0.01 |
| Death OR ROP Surgery | 41 | 43 | 48 | 38 | 36 | 32 | 38 | 28 | 38 | 14 | 0.94 (0.89, 0.98) * | 0.94 (0.88, 0.99) *  ^1^ 0.44 (0.39, 0.50)  ^3^ P<0.01  ^4^ 0.32 (0.17, 0.61)  ^7^ P=0.04 |
| Bronchopulmonary dysplasia | 37 | 26 | 28 | 27 | 27 | 32 | 29 | 32 | 31 | 21 | 1.00 (0.93, 1.09) | 1.00 (0.92, 1.08)  ^1^ 0.69 (0.57, 0.84) |
| Death or BPD | 62 | 59 | 63 | 54 | 58 | 57 | 54 | 47 | 56 | 31 | 0.96 (0.89, 1.03) | 0.95 (0.89, 1.03)  ^1^ 0.60 (0.50, 0.70) |
| Postnatal steroids for BPD | 9 | 12 | 12 | 18 | 17 | 18 | 37 | 24 | 24 | 16 | 1.16 (1.09, 1.23) * | 1.17 (1.10, 1.25) *  ^1^ 0.65 (0.58, 0.74)  ^4^ 0.52, (0.33, 0.82) |
| Medical treatment for PDA | 21 | 32 | 37 | 25 | 28 | 25 | 26 | 12 | 23 | 10 | 0.93 (0.88, 0.98) * | 0.90 (0.85, 0.96) *  ^1^ 0.70 (0.62, 0.79)  ^3^ P<0.01 |
| Necrotising enterocolitis (NEC) | 18 | 8 | 9 | 8 | 12 | 14 | 14 | 12 | 10 | £ | 0.96 (0.89, 1.03) | 0.96 (0.89, 1.04)  ^1^ 0.78 (0.67, 0.91)  ^3^ P<0.01  ^4^ 5.04 (2.84, 8.94) |
| Surgery for NEC, suspected NEC or bowel perforation | 9 | 6 | 5 | £ | £ | 10 | 5 | 8 | 9 | 5 | 1.04 (0.95, 1.14) | 1.06 (0.97, 1.17)  ^1^ 0.75 (0.61, 0.92)  ^4^ 7.12 (3.22, 15.76)  ^7^ P<0.01 |
| Death or NEC | 35 | 35 | 43 | 32 | 41 | 37 | 34 | 27 | 34 | 10 | 0.95 (0.91, 1.00) * | 0.94 (0.89, 1.00) *  ^1^ 0.62 (0.53, 0.73)  ^2^ 1.35 (1.00, 1.83)  ^3^ P=0.04  ^6^ 0.68 (0.54, 0.86) |
| Bacterial pathogen after day-3 of birth | 15 | 20 | 15 | 17 | 19 | 19 | 22 | 12 | 13 | 9 | 0.97 (0.92, 1.04) | 0.98 (0.92, 1.04)  ^1^ 0.74 (0.65, 0.85)  ^2^ 0.53 (0.37, 0.76)  ^3^ P=0.02 |
| Coagulase negative staphylococcal infection after day 3 of birth | 23 | 25 | 25 | 20 | 19 | 17 | 17 | 10 | 21 | 12 | 0.94 (0.88, 0.99) * | 0.92 (0.87, 0.98) *  ^1^ 0.77 (0.67, 0.87)  ^3^ P<0.01 |
| Any sepsis during stay | 37 | 39 | 38 | 34 | 35 | 37 | 41 | 21 | 34 | 24 | 0.97 (0.93, 1.02) | 0.98 (0.93, 1.03)  ^1^ 0.68 (0.58, 0.80)  ^2^ 0.00 (0.00, 0.61)  ^6^ 1.24 (1.00, 1.54)  ^7^ P<0.01 |
| Any late onset sepsis | 35 | 38 | 36 | 33 | 32 | 33 | 38 | 20 | 32 | 20 | 0.96 (0.91, 1.00) | 0.94 (0.89, 0.99) *  ^1^ 0.68 (0.61, 0.77)  ^2^ 0.66 (0.49, 0.89)  ^7^ P<0.01 |
| Death or sepsis during stay | 56 | 65 | 68 | 55 | 60 | 55 | 57 | 33 | 55 | 32 | 0.94 (0.90, 0.98) * | 0.93 (0.88, 0.98) *  ^1^ 0.52 (0.46, 0.58)  ^3^ P<0.01  ^4^ 0.66 (0.47, 0.93) |

Supplementary table 6: Detailed morbidity outcomes of infants in the study. Odds (95% CI) are for trend, calculated by logistic regression models with the year of birth as a continuous variable, and adjusted for gestational age at birth^1^, sex^2^, centre^3^, and outborn-status^4^, along with the interaction terms gestation*year of birth^5^, gestation*gender (1)^6^ and centre of care*outborn-status (1)^7^. $ = missing data was excluded before analysis, £ = values <5 suppressed to prevent accidental identification. ^!^ Superscript numbers indicate significant independent variable on adjusted analysis as numbered above.

| **Mortality of infants** | **Birth year (n)** | | | | | | | | | | **Unadjusted odds (95% CI) ^$^** | **Adjusted odds (95% CI) ^$ !^** |
| --- | --- | --- | --- | --- | --- | --- | --- | --- | --- | --- | --- | --- |
|  | **2007** | **2008** | **2009** | **2010** | **2011** | **2012** | **2013** | **2014** | **2015** | **2016** |  |  |
| Total | 105 | 96 | 101 | 95 | 94 | 101 | 111 | 87 | 97 | 61 |  |  |
| Delivery room deaths | £ | 6 | 10 | 8 | 9 | £ | £ | £ | 7 | £ | 0.94 (0.85, 1.04) | 1.01 (0.89, 1.15)  ^1^ 0.32 (0.24, 0.44)  ^3^ P<0.01 |
| Died after admission before discharge (absolute) | 25 | 28 | 28 | 20 | 23 | 24 | 22 | 17 | 21 | 9 | 0.94 (0.89, 0.99) * | 0.93 (0.87, 0.98) *  ^1^ 0.65 (0.54, 0.78)  ^2^ 1.40 (1.00, 1.95)  ^6^ 0.70 (0.54, 0.91) |
| Any death after live birth | 27 | 34 | 38 | 28 | 32 | 26 | 26 | 18 | 28 | 10 | 0.94 (0.89, 0.99) * | 0.93 (0.88, 0.99) *  ^1^ 0.60 (0.50, 0.71)  ^2^ 1.46 (1.06, 2.01)  ^3^ p<0.01  ^4^ 0.31 (0.15, 0.65)  ^6^ 0.71 (0.55, 0.90)  ^7^ P=0.04 |

Supplementary table 7: Detailed mortality outcomes of infants in the study. Odds (95% CI) are for trend, calculated by logistic regression models with the year of birth as a continuous variable, and adjusted for gestational age at birth^1^, sex^2^, centre^3^, and outborn-status^4^, along with the interaction terms gestation*year of birth^5^, gestation*gender (1)^6^ and centre of care*outborn-status (1)^7^. $ = missing data was excluded before analysis, £ = values <5 suppressed to prevent accidental identification. ^!^ Superscript numbers indicate significant independent variable on adjusted analysis as numbered above.

| **Characteristics of infants born at <28 weeks** | **Missing cases** | **Proportion of Total (%)** |
| --- | --- | --- |
| Sex | 0 | 0.0 |
| Birth Gestation | 0 | 0.0 |
| Birth weight | 0 | 0.0 |
| Head circumference at birth | 136 | 14.35 |
| Multiple birth | 0 | 0.0 |
| Outborn | 0 | 0.0 |
| Major birth defect | 0 | 0.0 |
| **Prenatal care** |  |  |
| Maternal race | 7 | 0.7 |
| Prenatal care | 1 | 0.1 |
| Maternal hypertension | 106 | 11.2 |
| Antenatal steroids used | 4 | 0.4 |
| Antenatal magnesium sulphate (from 2012) | 7 | 1.5 |
| Chorioamnionitis | 110 | 11.6 |
| Vaginal Delivery | 0 | 0.0 |
| **Resuscitation at delivery** |  |  |
| Oxygen during initial resuscitation | 1 | 0.1 |
| Face mask ventilation during initial resuscitation  (including CPAP till 2010) | 4 | 0.4 |
| Nasal CPAP during initial resuscitation  (from 2011, including nasal IMV and nasal SMV) | 50 | 5.3 |
| Any NIV during resuscitation (face-mask, CPAP) | 19 | 2.0 |
| Endotracheal tube ventilation during initial resuscitation | 1 | 0.1 |
| Epinephrine during initial resuscitation | 0 | 0.0 |
| Cardiac compressions during initial resuscitation | 1 | 0.1 |
| Surfactant during initial resuscitation | 0 | 0.0 |
| **Respiratory Care after resuscitation (delivery room deaths = 50)** |  |  |
| Surfactant at Any Time | 0 | 0.0 |
| Oxygen after Initial Resuscitation | 50 | 5.3% |
| Conventional Ventilation after Initial Resuscitation | 50 | 5.3% |
| High Frequency Ventilation after Initial Resuscitation | 50 | 5.3% |
| Any Mechanical Ventilation (MV, HFOV) after Initial Resuscitation | 50 | 5.3% |
| Nasal CPAP after Initial Resuscitation | 50 | 5.3% |
| Nasal IMV or Nasal SIMV (NIPPV) after Initial Resuscitation | 50 | 5.3% |
| High Flow Nasal Cannula after Initial Resuscitation | 50 | 5.3% |
| Any Non-Invasive Ventilation (nCPAP, NIPPV, HFNC) after Initial Resuscitation | 50 | 5.3% |
| **Outcomes* (delivery room deaths = 50)** |  |  |
| Death after livebirth and before discharge | 0 | 0.0 |
| Intra-ventricular haemorrhage (IVH) | 73 | 7.7 |
| Periventricular leukomalacia (PVL) | 67 | 7.1 |
| Severe CUSS abnormalities (Severe IVH OR PNV) | 72 | 7.6 |
| Death OR Severe CUSS (severe IVH/PVL) findings | 5 | 0.5 |
| Retinopathy of prematurity (ROP) | 331 | 34.9 |
| ROP Surgery | 4 | 0.4 |
| Death OR ROP Surgery | 4 | 0.4 |
| Bronchopulmonary Dysplasia (BPD) at 36-weeks | 547 | 57.7 |
| Steroids for BPD | 54 | 5.7 |
| Death OR BPD | 289 | 30.5 |
| Medical treatment for Patent Ductus Arteriosus (PDA) | 50 | 5.3 |
| Surgical ligation for PDA | 50 | 5.3 |
| Necrotising Enterocolitis (NEC) | 50 | 5.3 |
| NEC Surgery | 51 | 5.4 |
| Gastro-intestinal (GI) perforation | 51 | 5.4 |
| Death OR NEC | 0 | 0.0 |
| Any Sepsis | 50 | 5.3 |
| Any Early-onset Sepsis | 51 | 5.4 |
| Any Late Sepsis | 117 | 12.3 |
| Death OR Any Sepsis | 0 | 0.0 |

Supplementary table 8: Details of missing data.

| **Key outcomes** | **10-year Incidence n (%age of available data) ^$^** | **Missing Data n (%age)** | **Unadjusted odds (95% CI)** | **Adjusted odds (95% CI) ^!^** |
| --- | --- | --- | --- | --- |
| 24-27 weeks |  |  |  |  |
| Total Infants | 848 |  |  |  |
| Delivery room deaths | 17 (2.0) | 0 (0.0) | 0.82 (0.68, 0.99) * | 0.81 (0.66, 0.98) * ^1^ |
| Died after admission and before discharge | 179 (21.5) | 0 (0.0) | 0.95 (0.90, 1.01) | 0.95 (0.89, 1.01) |
| Any death after live birth | 196 (23.1) | 0 (0.0) | 0.94 (0.88, 0.99) * | 0.92 (0.86, 0.98) * ^1^ |
| Severe IVH OR PVL (Severe CUSS abnormalities) | 131 (16.1) | 32 (3.8) | 0.97 (0.90, 1.03) | 0.97 (0.90, 1.04) ^2, 3, 4, 5^ |
| Death OR Severe CUSS abnormalities | 279 (33.1) | 4 (0.5) | 0.95 (0.90, 1.00) | 0.95 (0.89, 1.00) * ^1, 2, 3^ |
| ROP surgery | 79 (9.6) | 21 (2.5) | 0.98 (0.90, 1.06) | 1.00 (0.91, 1.10) ^3, 5^ |
| Death OR ROP Surgery | 271 (32.1) | 4 (0.5) | 0.95 (0.90, 1.00) * | 0.95 (0.89, 1.00) ^1, 3, 4^ |
| Bronchopulmonary dysplasia | 266 (70.9) | 473 (55.8) | 1.00 (0.92, 1.08) | 0.99 (0.91, 1.08) |
| Death or BPD | 447 (79.4) | 285 (33.6) | 0.96 (0.89, 1.03) | 0.95 (0.88, 1.03) |
| Medical treatment for PDA | 212 (25.5) | 17 (2.0) | 0.93 (0.88, 0.99) * | 0.91 (0.85, 0.96) * ^3, 5^ |
| Necrotising enterocolitis (NEC) | 101 (12.2) | 17 (2.0) | 0.97 (0.90, 1.04) | 0.97 (0.89, 1.05) ^4, 5, 7^ |
| Death or NEC | 255 (30.1) | 0 (0.0) | 0.95 (0.91, 1.01) | 0.94 (0.89, 0.99) * ^1^ |
| Any early-onset sepsis | 28 (3.4) | 18 (2.1) | 1.09 (0.95, 1.25) | 1.11 (0.96, 1.27) |
| Any late-onset sepsis | 287 (36.8) | 69 (8.1) | 0.95 (0.90, 1.00) | 0.93 (0.88, 0.99) * ^1, 2, 7^ |
| Any sepsis during stay | 308 (37.1) | 17 (2.0) | 0.97 (0.92, 1.02) | 0.96 (0.91, 1.02) ^2, 5, 7^ |
| Death or sepsis during stay | 445 (52.5) | 0 (0.0) | 0.94 (0.90, 0.99) * | 0.94, (0.89, 0.99) * ^1, 3, 4^ |
| <24 weeks |  |  |  |  |
| Total Infants | 100 |  |  |  |
| Delivery room deaths | 33 (33.0) | 0 (0.0) | 1.03 (0.89, 1.19) | 1.55 (1.15, 2.10) * ^1, 3^ |
| Died after admission and before discharge | 38 (56.7) | 0 (0.0) | 0.90 (0.77, 1.04) | 0.82 (0.69, 0.99) * ^2, 3, 6^ |
| Any death after live birth | 71 (71.0) | 0 (0.0) | 0.91 (0.78, 1.07) | 1.00 (0.81, 1.22) ^1, 2, 3^ |
| Severe IVH OR PVL (Severe CUSS abnormalities) | 20 (33.3) | 40 (40.0) | 0.99 (0.82, 1.19) | 0.98 (0.80, 1.20) |
| Death OR Severe CUSS abnormalities | 77 (77.8) | 1 (1.0) | 0.89 (0.75, 1.06) | 0.95 (0.77, 1.17) ^1, 2, 3^ |
| ROP surgery | 15 (22.4) | 33 (33.0) | 0.87 (0.71, 1.07) | 0.80 (0.61, 1.04) ^2^ |
| Death OR ROP Surgery | 85 (85.0) | 0 (0.0) | 0.76 (0.62, 0.94) * | 0.76 (0.59, 0.99) * |
| Bronchopulmonary dysplasia | 24 (92.3) | 74 (74.0) | 1.55 (0.69, 3.49) | ! |
| Death or BPD | 94 (97.9) | 4 (4.0) | 1.70 (0.70, 4.12) | ! |
| Medical treatment for PDA | 27 (40.3) | 33 (33.0) | 0.94 (0.79, 1.11) | 0.85 (0.69, 1.04) ^3^ |
| Necrotising enterocolitis (NEC) | 5 (7.5) | 33 (33.0) | 0.78 (0.54, 1.14) | 0.75 (0.50, 1.12) |
| Death or NEC | 73 (73.0) | 0 (0.0) | 0.91 (0.77, 1.06) | 0.98 (0.80, 1.20) ^1, 2, 3^ |
| Any early-onset sepsis | 5 (7.5) | 33 (33.0) | 0.86 (0.62, 1.21) | 0.47 (0.20, 1.07) |
| Any late-onset sepsis | 30 (57.7) | 48 (48.0) | 0.99 (0.82, 1.19) | 0.96 (0.78, 1.18) |
| Any sepsis during stay | 32 (47.8) | 33 (33.0) | 1.07 (0.91, 1.26) | 1.02 (0.84, 1.24) ^2^ |
| Death or sepsis during stay | 91 (91.0) | 0 (0.0) | 0.80 (0.62, 1.03) | 0.84 (0.64, 1.11) |

Supplementary table 9: Sensitivity analysis for key outcomes by gestation groups (24-27 and <24 weeks gestation infants). Odds (95% CI) are for trend, calculated by logistic regression models with the year of birth as a continuous variable, and adjusted for gestational age at birth^1^, sex^2^, centre^3^ and outborn-status^4^, along with the interaction terms gestation*year of birth^5^, gestation*gender (1)^6^ and centre of care*outborn-status (1)^7^. ^$^ All analysis was conducted after excluding missing data, ! = odds could not be estimated due to high incidence. ^!^ Superscript numbers indicate significant independent variable on adjusted analysis as numbered above.

| **Key changes in Practice** | **10-year Incidence n (%age of available data) ^$^** | **Missing Data n (%age)** | **Unadjusted odds (95% CI)** | **Adjusted odds (95% CI) ^!^** |
| --- | --- | --- | --- | --- |
| 24-27 weeks |  |  |  |  |
| Total infants | 848 |  |  |  |
| Surfactant during initial resuscitation | 783 (92.3) | 0 (0.0) | 0.89 (0.81, 0.98) * | 0.90 (0.82, 0.99) * ^1, 3, 4^ |
| Surfactant at any time (absolute) | 820 (96.7) | 0 (0.0) | 0.85 (0.74, 0.98) * | 0.87 (0.76, 1.00) ^1, 3, 4^ |
| Any non-invasive ventilation during resuscitation (face-mask, CPAP) | 761 (90.7) | 9 (1.1) | 1.36 (1.23, 1.50) * | 1.34 (1.21, 1.48) * ^3, 7^ |
| Any non-invasive ventilation after resuscitation | 703 (84.6) | 17 (2.0) | 1.02 (0.95, 1.09) | 1.03 (0.96, 1.11) ^1^ |
| Any mechanical ventilation during resuscitation | 795 (93.9) | 1 (0.1) | 0.84 (0.75, 0.93) * | 0.84 (0.75, 0.94) * ^1, 2, 3, 4^ |
| Any mechanical ventilation after initial resuscitation | 811 (97.6) | 17 (2.0) | 0.79 (0.67, 0.95) * | 0.80 (0.68, 0.96) * ^1,^ |
| <24 weeks |  |  |  |  |
| Total infants | 100 |  |  |  |
| Surfactant during initial resuscitation | 72 (72.0) | 0 (0.0) | 1.04 (0.89, 1.22) | 0.83 (0.66, 1.05) ^1, 3^ |
| Surfactant at any time (absolute) | 74 (74.0) | 0 (0.0) | 1.01 (0.86, 1.18) | 0.94 (0.78, 1.13) ^1^ |
| Any non-invasive ventilation during resuscitation (face-mask, CPAP) | 81 (90.0) | 10 (10.0) | 0.85 (0.66, 1.10) | 0.79 (0.60, 1.05) |
| Any non-invasive ventilation after resuscitation | 30 (44.8) | 33 (33.0) | 1.10 (0.93, 1.30) | 1.02 (0.83, 1.25) ^1, 2^ |
| Any mechanical ventilation during resuscitation | 76 (76.0) | 0 (0.0) | 1.03 (0.87, 1.22) | 0.83 (0.65, 1.06) ^1^ |
| Any mechanical ventilation after initial resuscitation | 67 (100.0) | 33 (33.0) | ** | ** |

Supplementary table 10: Sensitivity analysis for key changes in practice of respiratory support by gestation groups (24-27 and <24 weeks gestation infants). Odds (95% CI) are for trend, calculated by logistic regression models with the year of birth as a continuous variable, and adjusted for gestational age at birth^1^, sex^2^, centre^3^ and outborn-status^4^, along with the interaction terms gestation*year of birth^5^, gestation*gender (1)^6^ and centre of care*outborn-status (1)^7^. ^$^ All analysis was conducted after excluding missing data, ** = odds could not be estimated due to high incidence. ^!^ Superscript numbers indicate significant independent variable on adjusted analysis as numbered above.

**References**

1. Shennan AT, Dunn MS, Ohlsson A, Lennox K, Hoskins EM. Abnormal pulmonary outcomes in premature infants: prediction from oxygen requirement in the neonatal period. *Pediatrics* 1988; **82**(4): 527-32.

2. Kliegman RM, Walsh MC. Neonatal necrotizing enterocolitis: pathogenesis, classification, and spectrum of illness. *Current problems in pediatrics* 1987; **17**(4): 213-88.

3. Papile LA, Burstein J, Burstein R, Koffler H. Incidence and evolution of subependymal and intraventricular hemorrhage: a study of infants with birth weights less than 1,500 gm. *J Pediatr* 1978; **92**(4): 529-34.

4. Aaberg TM, Abrams GW. Changing indications and techniques for vitrectomy in management of complications of diabetic retinopathy. *Ophthalmology* 1987; **94**(7): 775-9.
